# Retrospective and prospective: insights from a decade of anesthesiology trends for perioperative health care

**DOI:** 10.3389/fmed.2025.1547487

**Published:** 2025-04-28

**Authors:** Wenjie Xu, Zizheng Suo, Yinyin Qu, Bowen Zhou, Yuxiang Zheng, Cheng Ni

**Affiliations:** ^1^Department of Anesthesiology, National Cancer Center / National Clinical Research Center for Cancer / Cancer Hospital, Chinese Academy of Medical Sciences and Peking Union Medical College, Beijing, China; ^2^Department of Anesthesiology, Peking University Third Hospital, Beijing, China

**Keywords:** anesthesiology, keyword analysis, artificial intelligence, postoperative cognitive outcomes, perioperative brain health

## Abstract

This study analyzes research trends in anesthesiology over the past decade (2013–2023) by conducting a systematic keyword analysis of articles published in leading anesthesia journals, including Anesthesia, British Journal of Anesthesia, Anesthesiology, Journal of Clinical Anesthesia, and Anesthesia & Analgesia. Using the Bibliometrix software package (R version 3.5.6), we identified temporal patterns and journal-specific preferences in research themes. Early trends emphasized pain management, neuromuscular blockade, and perioperative physiology, while the COVID-19 pandemic shifted focus toward respiratory management and pandemic-related concerns. In recent years, research priorities have increasingly highlighted perioperative brain health, including postoperative cognitive dysfunction and delirium, alongside the integration of artificial intelligence to optimize perioperative care. These findings reflect the evolving landscape of anesthesiology, with a growing emphasis on mitigating postoperative complications and addressing the cognitive outcomes of surgical patients—issues of relevance to aging populations. By bridging gaps between research innovations and clinical application, this study underscores the importance of multidisciplinary collaboration to enhance patient outcomes and inform public health strategies in the perioperative setting.

## Introduction

Since the first public exhibition of successful general anesthesia in 1846, the field of anesthesiology has undergone over a century of development and transition, which is characterized by continuous progress, adaptation to new challenges, and the integration of new technologies to meet patient needs (1). The mission and research focus of anesthesiology has evolved from merely reducing perioperative mortality to enhancing patient comfort and long-term - functional outcomes (2). Given these advancements, our interest lies in identifying trends and innovative research in anesthesiology, as well as understanding the barriers to their implementation in clinical practice. Therefore, we conducted a comprehensive keyword analysis of articles published in top anesthesia journals over the past decade, to identify the potential gaps between clinical practice and research findings, and to explore strategies to bridge these gaps.

## Methods

”Keyword Plus” is a feature of bibliographic databases that automatically generates additional keywords for publications by extracting terms from the titles of cited references, rather than relying solely on author-provided keywords or those indexed from the title. This algorithmic approach broadens the indexing of relevant concepts and enhances the discoverability of articles by capturing implicit research themes that may not be explicitly stated in the original keywords (3). Due to the limitation of space and the requirement for a concise graphical presentation in Perspective articles, we selected five high-impact, comprehensive anesthesiology journals for inclusion. We systematically compiled and analyzed Keyword Plus data from all articles published in the past decade in top anesthesia journals including Anesthesia, British Journal of Anesthesia (BJA), Anesthesiology, Journal of Clinical Anesthesia (JCA), and Anesthesia & Analgesia. Bibliometrzix software (R version 3.5.6) was utilized for statistical analysis (4).

## Main results

**FIGURE 1:**
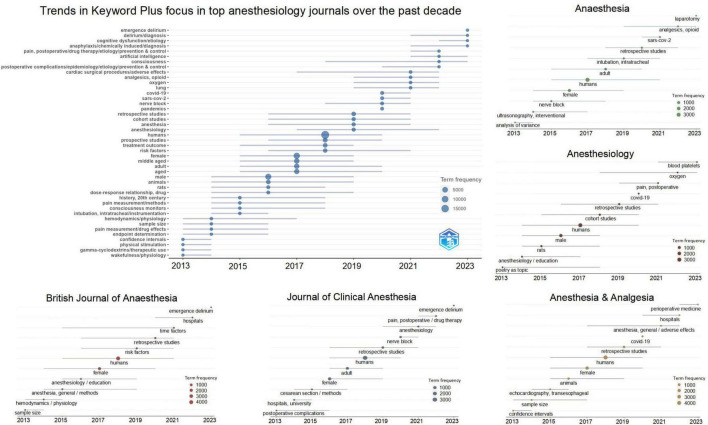
Evolution of Keyword Plus trends over the past decade (2013–2023), both overall and in individual top anesthesia journals. The data are presented both as an overall trend **(upper left corner)** and as individual trends within five top anesthesia journals: Anesthesia, Anesthesiology, British Journal of Anesthesia, Journal of Clinical Anesthesia, and Anesthesia & Analgesia. The horizontal lines represent the time span during which specific keywords were prevalent as hotspots in the literature. The size of the dots on the lines indicates the frequency of keyword occurrences within the years.

The results reveal the research trends within the field of anesthesiology over the past decade and highlight the research priorities of different journals. Specifically, the results are divided into three sections: the overall keyword characteristics across all journals, the overall trends and patterns of keywords across years, and the specific keyword characteristics of each journal. These findings are illustrated in Figure 1.

## Overall trends across all journals

A comprehensive analysis of keywords across all anesthesiology journals revealed several common themes. The frequent occurrence of “humans” underscores the continued dominance of clinical research in the field. High-ranking gender-related keywords (“female”, “male”) and age-related terms (“aged”, “middle-aged”, “adult”) highlight the growing emphasis on patient-specific factors in anesthetic care. The widespread presence of “retrospective studies” reflects the utility of large clinical datasets in anesthesiology research, while the inclusion of “prospective studies” and “cohort studies” points to increasing methodological diversity. Additionally, frequent mentions of “animal” and “rat” indicate sustained interest in basic and translational research, with animal models serving as essential tools for exploring anesthetic mechanisms and therapeutic potential.

## Overall trends and patterns across years

During the period from 2013 to 2015, the keywords “pain measurement,” “drug effects” and “gamma-cyclodextrins” indicate the emphasis on pain and neuromuscular blockade reversal studies (5). While keywords “wakefulness,” “consciousness monitors” and “hemodynamics” indicate the emphasis on perioperative physiology studies (6). From 2016 to 2019, the top keywords include “humans,” “female” and “middle-aged,” which do not have distinct characteristics. But the clinical practice has also improved during this period, due to the research advancements such as multimodal analgesia protocols, optimized circulatory strategies, etc. From 2020 to 2021, the top keywords “covid-19,” “sars-cov-2,” “oxygen,” “lung,” “pandemics” indicate that the anesthesia research has primarily focused on COVID-19 related topics. The pandemic has engaged the academic community, resulting in publications that aimed to enhance patient care and safety amidst the crisis. Other keywords include “cardiac surgical procedures,” “nerve block.” These keywords indicate that from 2013 to 2021, anesthesiologists consistently focused on perioperative anesthetic techniques and their innovations, particularly in the field of regional anesthesia.

From 2022 to 2023, anesthesia research focused on perioperative brain function and artificial intelligence (AI) related research, and the top keywords include “delirium,” “cognitive dysfunction,” “consciousness” and “artificial intelligence.” Another research focus is perioperative complications, and the keywords include “pain, postoperative”, “postoperative complications” and “anaphylaxis.” These recent researches on anesthesia journals aim to identify risk factors, preventive strategies, and therapeutic interventions to mitigate brain dysfunction following surgery (7). Additionally, the integration of AI into anesthesiology is becoming a significant area of interest, and have the potential to revolutionize anesthesia practices, from optimizing drug dosing, monitoring patient vital signs, and enhancing decision-making processes (8).

These changes in research trends can be attributed to multiple factors. From 2013 to 2015, progress in pharmacology and monitoring drove interest in pain and physiology. Between 2016 and 2019, clinical improvements such as enhanced recovery protocols and multimodal analgesia likely influenced the shift toward more practice-oriented research. The surge in COVID-19-related studies during 2020-2021 was driven by the pandemic’s global impact, as anesthesiologists played a key role in airway and critical care management (9). In 2022-2023, increasing awareness of postoperative neurocognitive complications and the rise of data-driven medicine contributed to the growing focus on brain function and AI (10). Together, these patterns reflect the influence of clinical needs, technological innovation, and global events on anesthesiology research priorities.

## Specific trends and patterns of each journal

The keyword analysis also shows the research preferences in different journals. For Anesthesia, the keywords “analgesics (2022),” “nerve block (2015)” and “ultrasonography (2014)” highlight its focus on the research of nerve block and pain management. And the top keywords also include “laparotomy (2023)” and “intubation (2019)”. For Anesthesiology, the recent top keywords include “blood platelets (2023),” “oxygen (2022)” and “postoperative pain (2021),” which indicate its preference in anesthesia research. In 2023, the top keyword for both BJA and JCA was “emergence delirium”, indicating a shared focus on postoperative brain function. Other unique keywords include “hemodynamics (2014)” for BJA, as well as “postoperative pain (2022),” “nerve block (2020),” “cesarean section (2015)” and “postoperative complications (2013)” for JCA, which indicate the preference of JCA on the research of postoperative pain and complications, especially for cesarean section. For Anesthesia & Analgesia, the recent top keyword “perioperative medicine (2023)” indicates the development direction of the field. The keywords “adverse effects (2021)” and “transesophageal echocardiography (2015)” signify its preference in anesthesia research.

## Discussion

In summary, this journey delineates the research trends in anesthesia journals during the past decade, which encompass a greater emphasis on brain function, AI, COVID-19, postoperative pain and complications. Anesthesiology plays a pivotal role in leading multidisciplinary teams, fostering collaboration across various departments. The pandemic and other global events have significantly influenced the academic landscape of this field.

Through the integration and analysis of keywords in recent years, the frequent occurrence of keywords such as “emergence delirium” and “cognitive dysfunction” suggests that future studies will continually focus on elucidating the mechanisms underlying postoperative cognitive disorders and delirium. Similarly, the prominence of keywords like “pain, postoperative” and “analgesics, opioid” indicates an ongoing effort to optimize postoperative pain management strategies and to identify safer and more effective analgesic approaches. Additionally, the presence of “artificial intelligence” highlights the emerging trend of integrating AI technologies into anesthesia practice, paving the way for advanced intraoperative monitoring, intelligent drug dosing, predictive analytics for postoperative complications, and clinical decision-support systems. Notably, AI is most frequently associated with ultrasonography in nerve block anesthesia, highlighting its applications in both image-guided techniques and personalized perioperative care. Other applications include leveraging machine learning to predict intraoperative hypotension, optimize anesthesia depth through EEG analysis, and enable early detection of postoperative complications. However, challenges such as data privacy concerns, algorithm transparency, and the need for clinical validation continue to hinder widespread implementation. Among the five journals, BJA and Anesthesia & Analgesia demonstrated the strongest focus on AI-related research, particularly in areas like machine learning and predictive modeling.

However, it is crucial to recognize that there are still less developed regions where both research and clinical practices lag behind global standards. Therefore, it remains essential to investigate whether these innovations can be effectively applied in such regions and to explore strategies for elevating the standard of care in less developed countries. Continued research is needed to address these disparities and ensure equitable advancements in anesthesiology worldwide.

## Data Availability

The original contributions presented in this study are included in this article/supplementary material, further inquiries can be directed to the corresponding author.
